# Antibacterial micro/nanomotors: advancing biofilm research to support medical applications

**DOI:** 10.1186/s12951-023-02162-0

**Published:** 2023-10-24

**Authors:** Zeyu Jiang, Lejun Fu, Chuang Wei, Qinrui Fu, Shuhan Pan

**Affiliations:** 1 Department of Emergency Medicine, The Affiliated Hospital of Qingdao University, Qingdao University, Qingdao, 266003 China; 2 Institute for Translational Medicine, The Affiliated Hospital of Qingdao University, College of Medicine, Qingdao University, Qingdao, 266021 China; 3 School of Chemistry and Materials Science, Anhui Normal University, Wuhu, 230022 China

**Keywords:** Micro/nanomotors, Multi-drug resistant bacteria, Biofilm, Antibacterial therapy

## Abstract

Multi-drug resistant (MDR) bacterial infections are gradually increasing in the global scope, causing a serious burden to patients and society. The formation of bacterial biofilms, which is one of the key reasons for antibiotic resistance, blocks antibiotic penetration by forming a physical barrier. Nano/micro motors (MNMs) are micro-/nanoscale devices capable of performing complex tasks in the bacterial microenvironment by transforming various energy sources (including chemical fuels or external physical fields) into mechanical motion or actuation. This autonomous movement provides significant advantages in breaking through biological barriers and accelerating drug diffusion. In recent years, MNMs with high penetrating power have been used as carriers of antibiotics to overcome bacterial biofilms, enabling efficient drug delivery and improving the therapeutic effectiveness of MDR bacterial infections. Additionally, non-antibiotic antibacterial strategies based on nanomaterials, such as photothermal therapy and photodynamic therapy, are continuously being developed due to their non-invasive nature, high effectiveness, and non-induction of resistance. Therefore, multifunctional MNMs have broad prospects in the treatment of MDR bacterial infections. This review discusses the performance of MNMs in the breakthrough and elimination of bacterial biofilms, as well as their application in the field of anti-infection. Finally, the challenges and future development directions of antibacterial MNMs are introduced.

## Introduction

Bacterial infections are a long-term threat to human health, causing millions of deaths every year, and have become a global public health problem [1–3]. Since the discovery of penicillin in 1928, antibiotics have been proved to be the most effective way to treat bacterial infections [4, 5]. However, traditional antibiotics are far from meeting the clinical demand due to the increase of multi-drug resistant (MDR) bacteria caused by antibiotic abuse [6–9]. Over 80% of antibiotic resistance is associated with the formation of bacterial biofilms [10], which are surface-associated bacterial communities surrounded by extracellular polymeric substances (EPS). These biofilms serve as a barrier to hinder the penetration and diffusion of antibiotics [11], allowing bacteria to be almost 1000-fold more resistant to conventional antibiotic treatments [12]. Therefore, addressing the challenge posed by biofilm formation is of great significance for the treatment of bacterial infections.

In recent years, the anti-bacterial properties of nanomaterials have garnered significant attention. These properties arise from the interactions between nanomaterials and bacteria, which result in the destruction of cell structures and eventual bacterial death. The physical antibacterial properties of nanomaterials, such as morphology, optics, thermology, and mechanics, play a crucial role in this process [13, 14]. For instance, the nanostructure on the surface of cicada wings exhibits a great bactericidal effect on *Pseudomonas aeruginosa (P. aeruginosa)* through physical cutting [15]. Furthermore, nanomaterials that release specific metal ions, such as Ag^+^, Cu^2+^, and Zn^2+^, can disrupt microbial protein function, impair membrane function, and interfere with nutrient absorption, leading to bacterial death [16]. Nanoscale antibacterial materials also facilitate the penetration of biofilms, which is advantageous for the treatment of MDR bacterial infections. Nevertheless, the passive penetration of biofilms remains slow, inefficient, and inadequate, failing to completely eradicate resistant bacteria. In light of the rapid advancements in nanotechnology and nanomaterials, micro/nanomotors (MNMs) offer a novel approach for treating MDR bacteria by autonomously reaching difficult-to-access sites, including deep biofilms, and performing specific tasks such as antibiotic action [17, 18]. MNMs can be customized in terms of composition, structure, and functionality to achieve precise motion control and drug delivery in complex physiological environments. Initially, MNMs were employed as drug carriers to deliver antibiotics and antibacterial substances [19–22]. Although they exhibit excellent delivery efficiency in breaking through biological barriers such as the gastric mucosal barrier, biofilm barrier, and blood–brain barrier, this method proved ineffective against antibiotic/antimicrobial peptide insensitive bacteria. Therefore, researchers developed non-antibiotic antibacterial phototherapy, including techniques like photothermal therapy (PTT), photodynamic therapy (PDT), and photocatalysis therapy (PCT), all of which induce bacterial apoptosis through photothermal transformation and the generation of cytotoxic reactive oxygen species (ROS) [23–25]. Nevertheless, the limited action radius of the photothermal effect and ROS has hindered their antibacterial efficacy and clinical application [26, 27]. This limitation is overcome by MNMs, which extend the action radius and enhance the antibacterial effect, providing a solution to this obstacle [28, 29]. Additionally, antibacterial MNMs have been employed to address two challenges in the field of anti-infection: superficial tissue infections and implant infections [30].

**Fig. 1:**
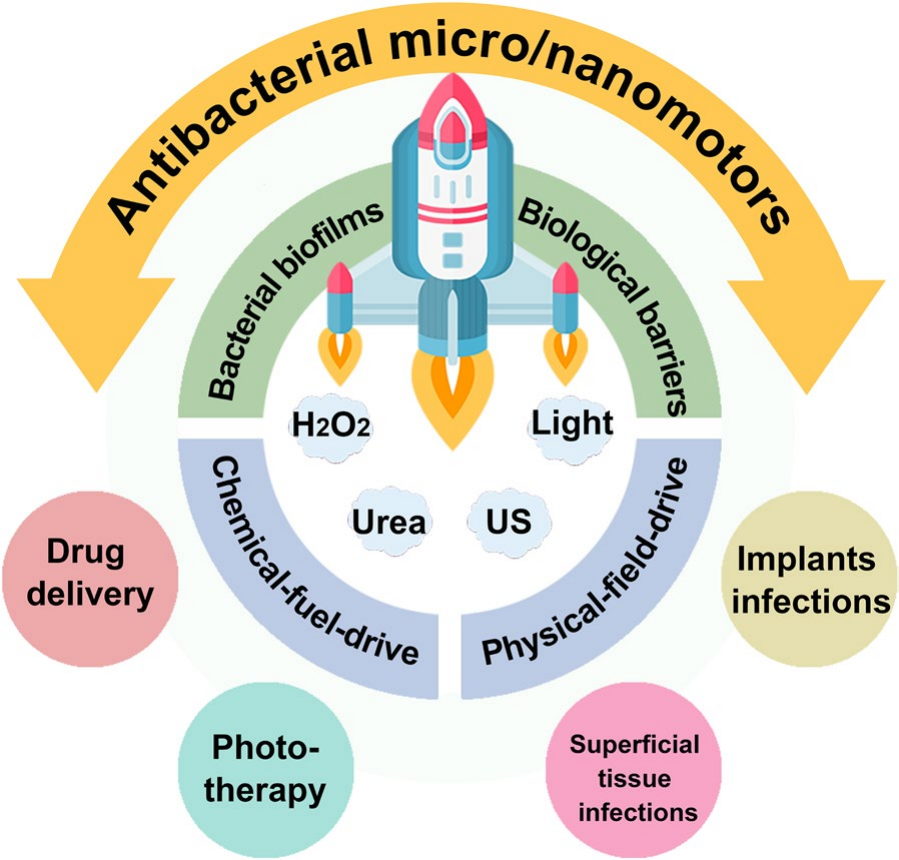
Schematic illustration of micro/nanomotors for breakthrough biofilm and antibacterial therapy

In this review, we first introduce the effect of MNMs in breakthrough biofilm, focusing on its composition, driving mode, power source and performance. Secondly, we review antibacterial strategies based on MNMs, including drug delivery and phototherapy, and highlight their principles and efficacy. And then, the application of MNMs in superficial tissue infections and implant infections were introduced, and its advantages in this field are emphasized (Fig. 1). Finally, opportunities and challenges in the design and preparation of antibacterial MNMs are provided, in order to promote its early application in clinical practice and benefit patients.

## Breakthrough biofilms

**Fig. 2:**
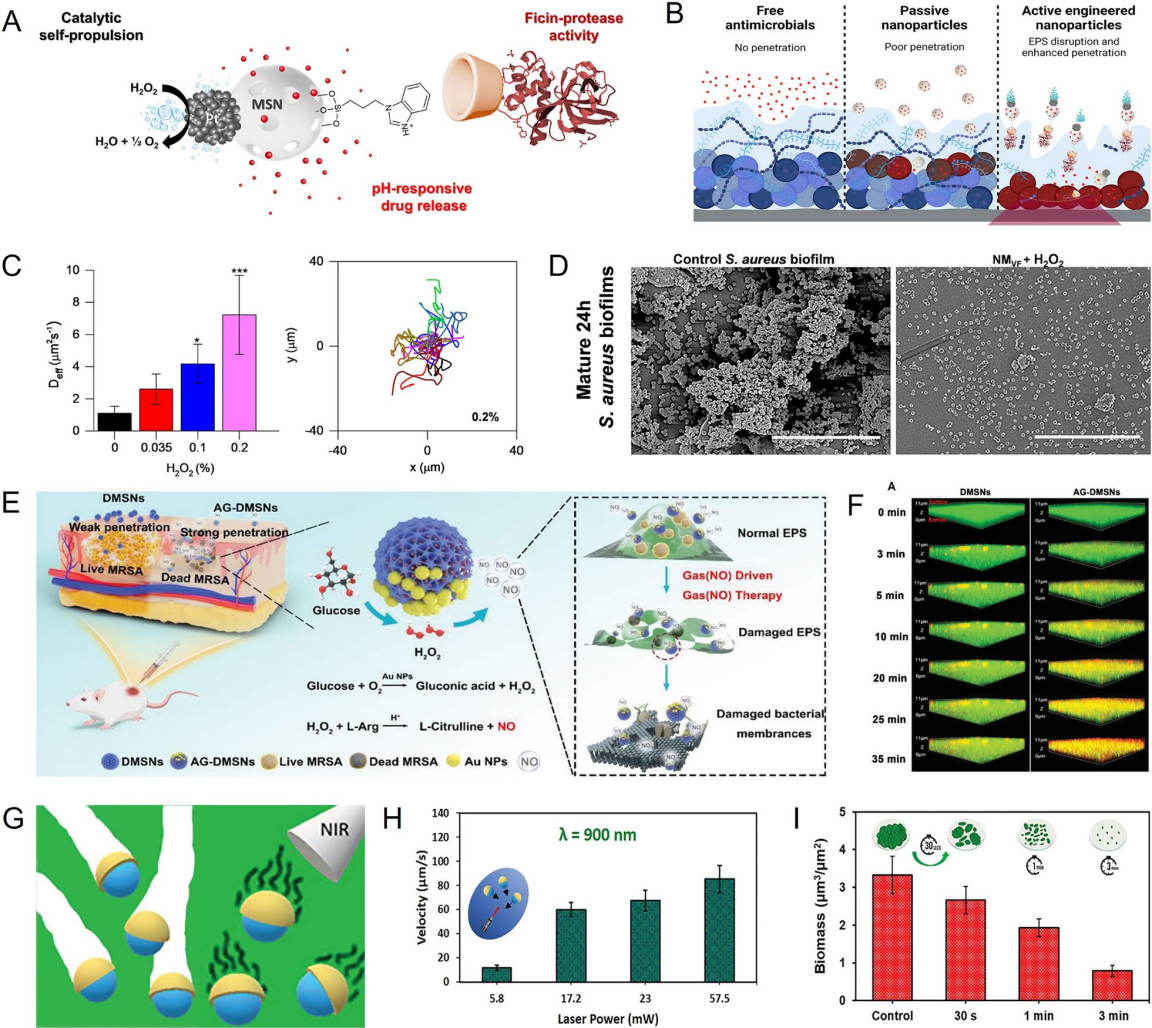
Breakthrough biofilm strategy based on MNMs.** A** Schematic illustration of the structure of Janus Pt − MSN. **B** Resistance of biofilms to different antimicrobial strategies. **C** The diffusion of Janus Pt-MSN at different H_2_O_2_ concentrations. **D** Disruption of *S. aureus* biofilms in different treatment groups. **E** Schematic illustration of NO-driven AG-DMSNs penetrating MRSA biofilm. **F** Penetration of DMSNs and AG-DMSNs into MRSA biofilms. **G** Schematic illustration of SiO_2_/Au nanomotors propulsion and biofilm removal under NIR irradiation. **H** The average velocity of SiO_2_-Au nanomotors as a function of the NIR laser power. **I** Biofilm content after treatment with SiO_2_-Au nanomotors under NIR irradiation. **A–D** Reprinted with permission [48]. Copyright 2023, American Chemical Society. **E–F** Reprinted with permission [49]. Copyright 2022, Wiley–VCH. **G-I** Reprinted with permission [55]. Copyright 2023, Wiley–VCH

There is an urgent need to develop MNMs and novel therapies that can effectively eradicate bacteria and break through biofilms [31, 32]. MNMs movement is driven by a key factor that not only affects their breakthrough performance on biofilms but also determines their biological function [33–35]. Based on their driving mechanism, MNMs can be classified into two categories: chemical-fuel-driven MNMs and external physical-fields-driven MNMs [36–47]. Chemical-fuel-driven MNMs utilize biocompatible components present at the infection site as fuel. These MNMs modify specific enzymes on their surface, resulting in the production of gas or chemical gradients that facilitate biofilm penetration. In a study conducted by Ramon et al., a pH-responsive H_2_O_2_-driven MNMs (Janus Pt-MSN) was developed (Fig. 2A) [48]. Platinum nanodendrites (PtNDs), the driving element, used the high concentration of H_2_O_2_ at the infection site to catalyze the production of O_2_, propelling the MNMs to break through bacterial biofilm (Fig. 2B). Moreover, the ficin enzyme modified on the MNMs hydrolyzed EPS, which effectively destroys the biofilm and exposes the bacteria. At the same time, the acidic biofilm microenvironment triggered the release of vancomycin for antibiotic delivery. The experiment demonstrated that the nanomotors exhibited significant diffusion coefficients (7.22 ± 2.45 μm^2^ S^−1^) compared to the control group (1.11 ± 0.42 μm^2^ S^−1^), and achieved 82% of biofilm disruption and 96% reduction in *staphylococcus aureus (S. aureus)* even at relatively low H_2_O_2_ concentrations (Fig. 2C). Figure 2D illustrates the successful performance of the nanomotors. Antibiotics are not only difficult to eliminate chronic infections caused by biofilms but may also promote the emergence of drug-resistant strains. Therefore, the development of MNMs and innovative therapies is crucial in addressing these challenges.

The limited sustained effects of MNMs, due to low concentrations of H_2_O_2_ and limited antibiotic loading, necessitate the design of a cascade catalytic and non-drug antibacterial MNMs. For example, Liu et al. developed AG-DMSNs, a self-catalytic asymmetry nanomotor, to eradicate biofilm using the nitric oxide (NO) generated by the cascade reaction, thereby achieving effective antibacterial therapy (Fig. 2E) [49]. AG-DMSNs, synthesized by loading L-arginine (L-Arg) and gold nanoparticles (AuNPs) on dendritic mesoporous silica nanoparticles (DMSNs), possess properties that simulate glucose oxidase (GOx). Consequently, AG-DMSNs can consume glucose to produce H_2_O_2_, which in turn oxidizes L-Arg and leads to NO production. The resulting NO not only induces autonomous movement to penetrate the biofilm deeply but also eradicates the biofilm and kills embedded bacteria by generating oxidative byproducts (nitrous oxide and peroxynitrite), which cause bacterial membrane destruction, DNA fragmentation, and protein dysfunction. In the methicillin-resistant *S. aureus* (MRSA) biofilm model, AG-DMSNs were observed at a depth of 7.1 µm within 35 min of incubation, while DMSNs diffused only to a depth of 2.2 µm, indicating the excellent penetration and motion capabilities of AG-DMSNs (Fig. 2F). Furthermore, AG-DMSNs achieved 99% anti-biofilm efficiency and reduced the bacterial burden by four orders of magnitude in a mouse wound model, highlighting the significant efficacy of nanomotors in combating drug-resistant bacteria.

On the other hand, external physical-fields-driven MNMs rely on energy input from external physical fields, such as light, magnetic, electric, and ultrasonic fields, to obtain kinetic driving force. This mechanism allows MNMs to effectively avoid the limitations of chemical fuels [50]. Among them, near-infrared (NIR) driven nanomotors are considered ideal candidates for external physical field propulsion [51]. Unlike chemical propulsion, which relies on chemical fuels, NIR-driven MNMs obtain kinetic driving force from an external field. This allows them to effectively avoid the limitations associated with chemical fuels [52]. Moreover, NIR-driven MNMs have high tissue penetration capacity, are easily obtainable, and cause minimal harm to the body [53, 54]. Boisen et al. developed a self-propelled mesoporous SiO_2_/Au nanomotor driven by NIR for the eradication of *P. aeruginosa* biofilm (Fig. 2G) [55]. This SiO_2_/Au nanomotor, with an asymmetrical structure, exhibits light-driven motion based on the thermophoresis mechanism, owing to the photosensitive properties of gold (Au). Interestingly, the SiO_2_/Au nanomotor is capable of remotely adjusting its speed by manipulating the power of the applied laser. At a laser power of 57.5 mW, the nanomotors can achieve speeds of up to 86 μm s^−1^, a speed that surpasses those attained in previous studies (Fig. 2H). The deep penetration of SiO_2_/Au nanomotors through the biofilm matrix mechanically destroyed the biofilm, resulting in the eradication of *P. aeruginosa* biofilm by over 70% in just 3 min (Fig. 2I). This demonstrated the exceptional controllability and efficiency of NIR-driven nanomotors. These results demonstrated that both endogenous substrate-driven nanomotors and exogenous propulsion nanomotors have excellent performance in breaking through and eliminating bacterial biofilms.

## Antibacterial strategy based on MNMs

In the last 5 years, antibacterial strategies based on MNMs have rapidly developed due to their excellent performance in overcoming biofilms [56]. The transformation of these strategies has shifted from simply improving antibacterial drug delivery, which includes antibiotics, antibacterial ions, and antimicrobial peptides, to incorporating new antibacterial treatments like photothermal therapy, photodynamic therapy, and sonodynamic therapy.

### Drug delivery based on MNMs

**Fig. 3:**
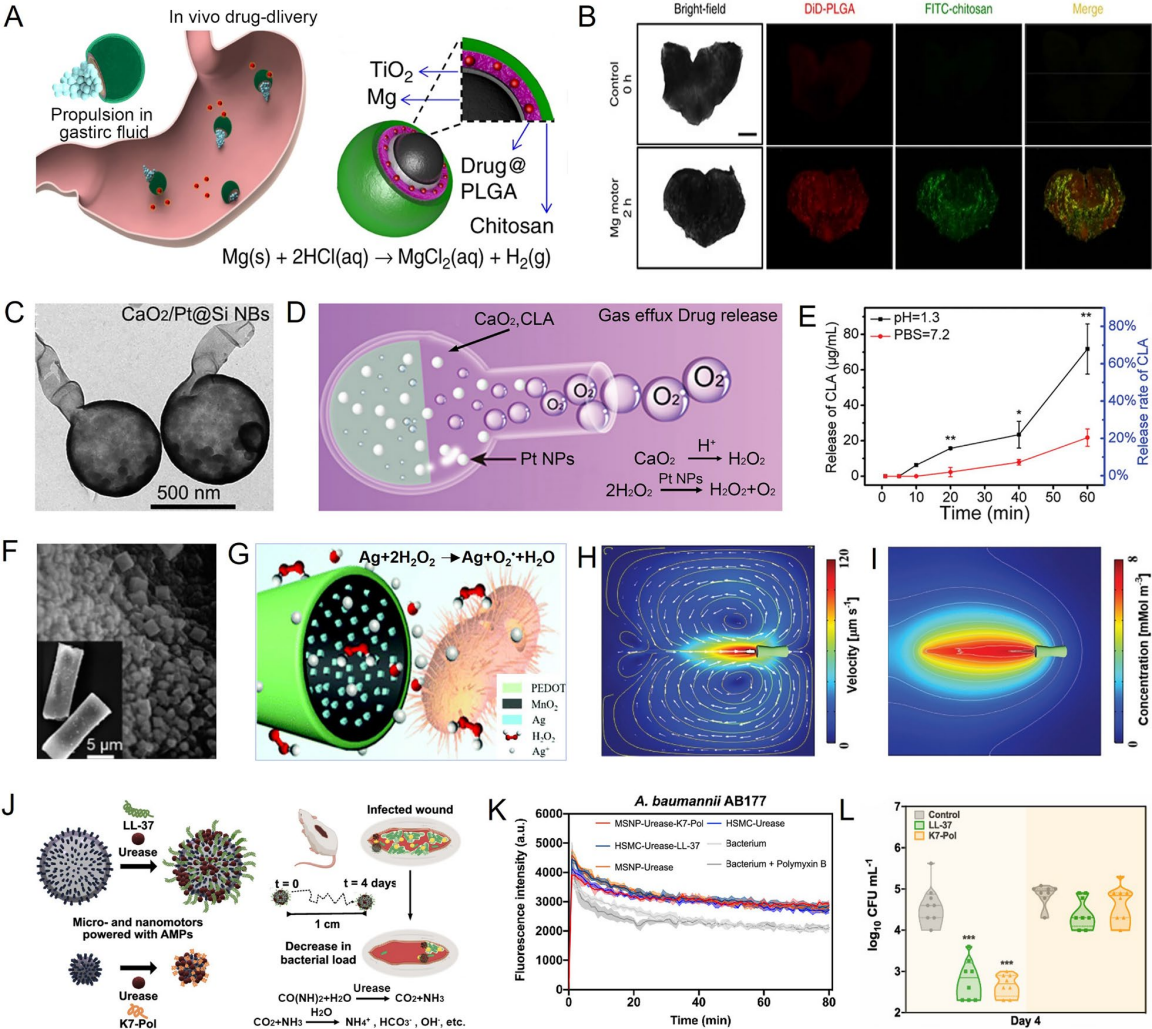
Drug delivery based on MNMs.** A** Schematic illustration of propulsion and drug delivery of the Mg-based micromotors. **B** Bright-field and fluorescence images of the stomach wall in control group and treatment group. **C** TEM images of CLA/CaO_2_/Pt@Si NBs. **D** Schematic illustration of propulsion and drug delivery of CLA/CaO_2_/Pt@Si NBs. **E** Release of CLA from the nanomotors in acidic and neutral pH. **F** SEM images of PEDOT/MnO_2_@Ag micromotors. **G** Schematic illustration of drive and antibacterial mechanism of PEDOT/MnO_2_@Ag micromotors. **H** Simulation of the velocity field. **I** Concentration distribution of Ag^+^. **J** MNMs coated with AMPs for the treatment of bacterial infections. **K** The depolarization efficiency of functionalized and nonfunctionalized MNMs. **L** Bacterial counts in different treatment groups. **A, B** Reprinted with permission [57]. Copyright 2017, Springer Nature. **C–E** Reprinted with permission [58]. Copyright 2021, Wiley–VCH. **F–I** Reprinted with permission [62]. Copyright 2020, The Royal Society of Chemistry. **J–L** Reprinted with permission [68]. Copyright 2022, American Chemical Society

Wang et al. developed a magnesium (Mg)-based micromotor loaded with the antibiotic clarithromycin (CLR) as a carrier for efficient delivery to treat *Helicobacter pylori (H. pylori)* infections (Fig. 3A) [57]. The micromotor had a Janus core–shell structure with the Mg microparticles as the core, asymmetrically distributed TiO_2_ as the inner shell, CLR-loaded poly(lactic-co-glycolic acid) (PLGA) layer, and an outer chitosan layer. In the stomach acid environment, the Mg core reacted with gastric acid to produce hydrogen (H_2_), which propelled the micromotor, allowing it to penetrate the gastric mucus and increase retention in the mucosal layer (Fig. 3B). This active drug delivery system showed significant benefits compared to free drug delivery, with the micromotor increasing drug delivery and reducing *H. pylori* burden. To further enhance drug loading, Han et al. designed a nanomotor with a large chamber and narrow opening (CLA/CaO_2_/Pt@Si NBs) (Fig. 3C) [58]. This nanomotor consisted of silica nanobottles (Si NBs) loaded with clarithromycin (CLA), calcium dioxide nanoparticles (CaO_2_ NPs), and platinum nanoparticles (Pt NPs). In the stomach cavity, CaO_2_ consumed protons (H^+^) and generated hydrogen peroxide (H_2_O_2_), catalyzed by Pt NPs to produce O_2_ (Fig. 3D). The resulting oxygen bubbles not only propelled the nanomotors but also facilitated drug release. The nanomotors demonstrated excellent drug loading and release rates, with a 10.52 wt% loading rate and 68.2% release rate for CLA (Fig. 3E). In mice experiments, the *H. pylori* burden was significantly lower (2.6 orders of magnitude) in the group treated with acid-powered nanomotors, demonstrating their effectiveness in drug delivery and killing bacteria.

In addition to delivering antibiotics, MNMs are also used for the delivery of antibacterial ions and antimicrobial peptides (AMPs), which overcomes the shortcomings of bacterial drug resistance and improves antibacterial performance and efficiency [59, 60]. For example, silver (Ag) exhibits broad-spectrum antibacterial activity against bacterial species such as *Escherichia coli* (*E. coli*), *Bacillus subtilis (B. subtilis)* and *S. aureus* [61]. Gu et al. prepared a micromotor (PEDOT/MnO_2_@Ag) by polymerizing the poly(3,4-ethylenedioxythiophene)(PEDOT) layer and the cathodic co-electrodeposition of MnO_2_ and Ag to treat *E. coli* infection through the bactericidal action of Ag^+^ (Fig. 3F, G) [62]. Due to the synergistic catalytic reaction of MnO_2_ and Ag to H_2_O_2_, the PEDOT/MnO_2_@Ag micromotor fueled by only 0.2% H_2_O_2_ achieved efficient motion with a velocity of up to 122 μm s^−1^ (Fig. 3H) and maintained 92% antibacterial performance from the on-the-fly release of Ag^+^ ions (Fig. 3I). This provides a good reference for the application of antibacterial materials in treating drug-resistant bacteria. In addition to bactericidal metal ions, AMPs have emerged as promising antibacterial agents due to their amphipathic character, which enables their interaction with and subsequent disruption of bacterial membranes [63–65]. However, the clinical translation of AMPs was limited by their limited bioavailability, susceptibility to enzymatic degradation, and low penetrability toward the target infections [66, 67]. Thus, efficient delivery methods are required to help these molecules reach their target area. Nunez et al. prepared a urea-fueled enzymatic nanomotor by loading urease and cationic AMPs (LL-37 and K7-Pol) onto silica-based NPs to actively navigate toward the infection site (Fig. 3J) [68]. The movement of the nanomotors is driven by the electric field force generated by the release of ion products through the decomposition of urea. When the urease-nanomotors reach the infection site, the initial electrostatic interactions between the negatively charged bacterial membranes and the positively charged AMPs prompt them to target bacteria and trigger the depolarization of bacterial membranes (Fig. 3K), resulting in bacteria death. In a murine infection model, the AMPs-modified nanomotors demonstrated autonomous propulsion, reducing *Acinetobacter baumannii* (*A. baumannii)* infections by up to 3 orders of magnitude, while free peptides were unable to exert antimicrobial activity at a distance from the initial administration site (Fig. 3L). These results demonstrated MNMs as drug carriers can not only reach otherwise inaccessible area, but also expand the distribution range of drugs to achieve better antibacterial effects.

### Phototherapy based on MNMs

Due to the existence of biofilms, antibiotics and other contact-type bactericidal materials unable effectively act on bacteria in the infected area, making the antibacterial efficiency significantly reduced. This has led researchers to make efforts in developing efficient, non-toxic and non-antibiotic new antibacterial agents and advanced treatment technologies [69, 70]. Phototherapy is a promising approach to treat bacterial infections due to its spatiotemporal selectivity, non-invasiveness, and minimal side effects [71]. In the 1920s, ultraviolet (UV) light with DNA damaging properties was used to sterilize the air and it has been effective against bacteria [72]. However, UV light is cytotoxic and poorly suited for tissue penetration, which hinders its application in vivo. In recent years, based on biosafe NIR, researchers have explored other forms of phototherapy such as photothermal therapy (PTT), photodynamic therapy (PDT), and photocatalytic therapy (PCT) for the treatment of bacterial infections [73–75]. Because NIR (700–1400 nm) is an electromagnetic wave with low frequency and weak energy, which does not cause direct harm to the human body. These therapies have shown promising application prospects as they convert light into heat energy or induce the production of ROS to cause bacterial apoptosis [76, 77].

**Fig. 4:**
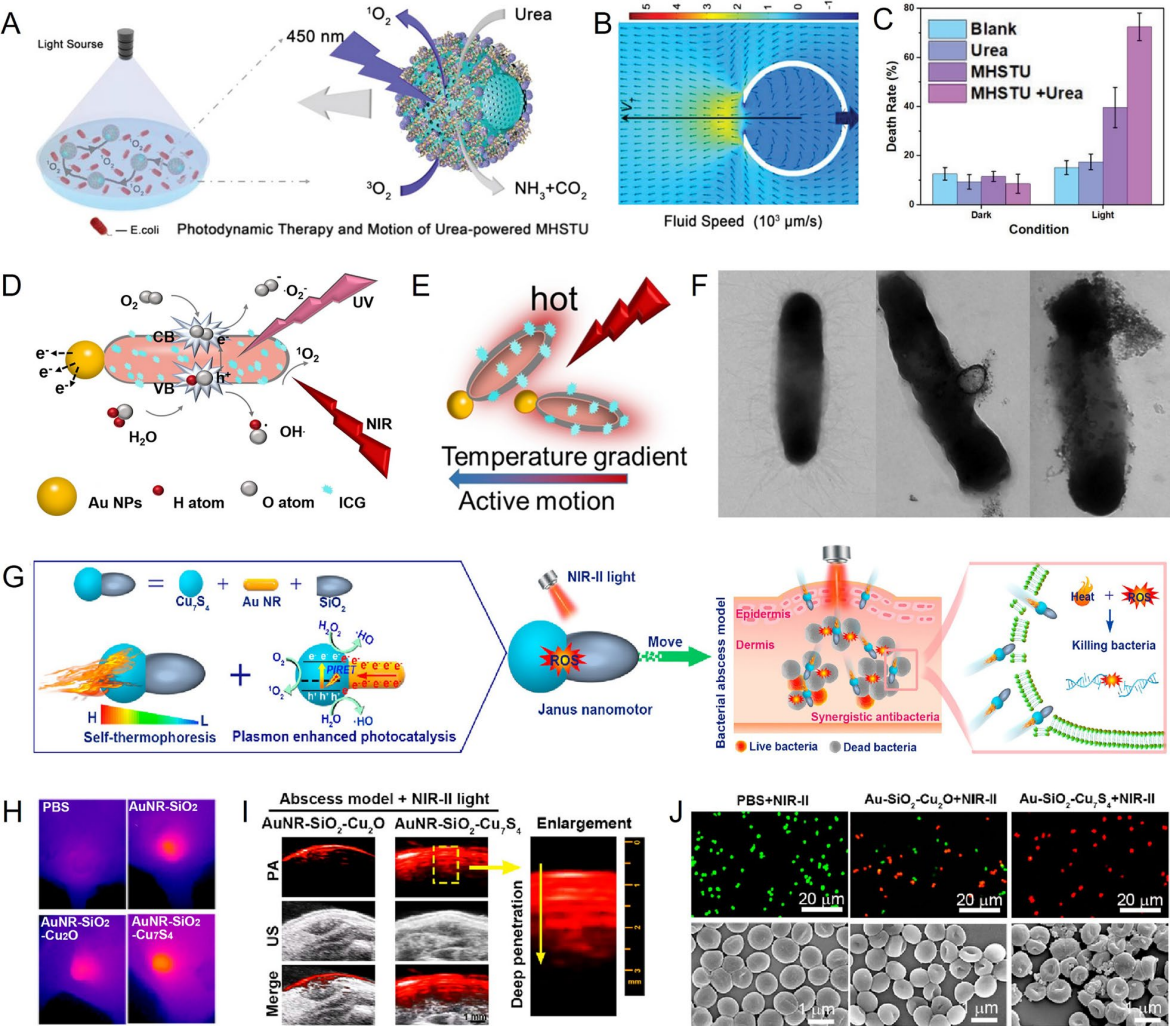
Phototherapy based on MNMs.** A** Schematic illustration of the urease-driven MHSTU for photodynamic antibacterial therapy. **B** Flow field of MHSTU powered by urea. **C** The death rates of *E. coli* after different treatments. **D** The mechanism of Au@ZnO@SiO_2_-ICG collaboratively enhanced the PTT/PDT antibacterial treatment. **E** The propulsion mechanism of Au@ZnO@SiO_2_-ICG under NIR irradiation. **F** TEM images of *E. coli* under NIR irradiation at different times. **G** Design and application of AuNR-SiO_2_-Cu_7_S_4_ nanomotors. **H** Thermal images undergoing different treatments. **I** Photoacoustic images and ultrasonic images of the abscess site in different treatments. **J** live/dead stained images of MRSA and bacterial morphology observed by SEM after different treatments. **A–C** Reprinted with permission [80]. Copyright 2019, WILEY–VCH. **D–F** Reprinted with permission [81]. Copyright 2022, The Royal Society of Chemistry. **G–J** Reprinted with permission [88]. Copyright 2023, American Chemical Society

Despite these advantages, the action radius of ROS/heat energy in phototherapy is often limited and the bacterial membrane can naturally block foreign substances, impeding the effectiveness of treatment. Consequently, researchers have turned to MNMs as a means to address this challenge and have achieved remarkable therapeutic effects in the field of antibacterial phototherapy [78, 79]. Ma et al. prepared a urease-driven micromotor (MHSTU) for highly efficient antibacterial PDT [80]. The micromotor was based on hollow mesoporous SiO_2_ (mSiO_2_) microspheres loaded with 5,10,15,20-tetrakis(4-aminophenyl)porphyrin (TAPP, a photosensitizer), urease and magnetic Fe_3_O_4_ NPs (Fig. 4A). MHSTU achieved phoretic motion driven by enzymatic reaction and direction of motion was directed by applying an external magnetic field, which significantly expanded the coverage area approximately 10 times (Fig. 4B). Under 450 nm light irradiation (14.2 mW cm^−2^), MHSTU generated cytotoxic singlet oxygen (^1^O_2_), resulting in a 72.5% *E. coli* kill rate*.* Importantly, compared with the non-fuel group, the MHSTU group exhibited a 20% increase in ^1^O_2_ yield and a 32.9% increase in bactericidal rate, attributed to the self-propelled motor’s ability to capture a wider range of O_2_ and expand ROS distribution (Fig. 4C). However, PDT alone was insufficient for achieving the desired bactericidal effect. Hence, Mao et al. developed multifunctional Janus nanomotors (Au@ZnO@SiO_2_-ICG) to achieve synergistic bacteria killing through the combination of PTT/PDT (Fig. 4D) [81]. The nanomotors were prepared by Au seed mediated nucleation and ZnO growth, following by coating with a SiO_2_ thin layer on the ZnO part and loading with the photosensitizer indo cyanine green (ICG). Under the irradiation of NIR light (808 nm), the nanomotors, primarily consisting of NPs and ICG, produced an asymmetric PTT and photothermal effect, leading to a self-heating force-driven speed of up to 5.6887 μm s^−1^ (Fig. 4E). Simultaneously, NIR light triggered cytotoxic ROS production of ICG, enhancing PDT produced by UV irradiation of ZnO. When *E. coli* bacteria were incubated with Au@ZnO@SiO_2_-ICG for two hours and exposed to NIR light and UV light, the nanomotors successfully penetrated the bacterial membrane, resulting in irreparable cracking and complete destruction of the bacterial morphology (Fig. 4F). Consequently, the treatment achieved an almost 100% bactericidal rate.

The limited in vivo application of nanomotors that rely on NIR-I and UV to kill bacteria is due to the adverse effects of UV on normal cells and poor tissue penetration of NIR-I [82, 83]. However, NIR-II light-mediated PTT and PCT offer promising in vivo antibacterial effects due to the preferred synergistic antimicrobial efficiency and the advantages of NIR-II light, such as deep penetration, low optical absorption, minimal scattering from tissue, and maximum permissible exposure [84–87]. A study by Song et al. reported the development of multifunctional nanomotors (AuNR-SiO_2_-Cu_7_S_4_) driven by NIR-II light (1064 nm) [88]. These nanomotors exhibited photocatalytic and photothermal synergistic antibacterial activities, rapid motion properties, and controllable, safe, highly efficient, and thorough bacteria-killing capabilities (Fig. 4G). When exposed to NIR-II light, a distinct thermal gradient was formed across the nanomotors, resulting in an enhanced local photothermal field close to the AuNR-Cu_7_S_4_ interface (Fig. 4H). This enabled the nanomotors to be actively driven via the self-thermophoresis effect at a speed of approximately 9.8 μm/s. Additionally, photocatalytic reactions produced a large number of ROS. The strong NIR-II photoacoustic (PA) imaging signal of the nanomotors, due to their strong optical absorption in the NIR-II window, can be used to observe penetration effects and guide the real-time treatment of bacterial infections (Fig. 4I). The experimental results demonstrated that the nanomotors exhibited an antibacterial efficiency of 98.3% in vitro and 97.8% in vivo (Fig. 4J). Importantly, irradiation of NIR-II light did not have adverse effects on normal cells and tissues. In conclusion, the development of nanotechnology has made it possible to combine MNMs with new therapeutics as antibacterial agents, overcoming the shortcomings of antibiotic resistance and limited drug loading. In particular, the mechanism of PTT is that NIR interacts with MNMs to produce thermal effects, thus causing bacterial pyrolysis, which depends on the photothermal conversion efficiency of MNMs and its enrichment at the infected site [84]. On the other hand, PCT and PDT rely on ROS production, which puts higher demands on local oxygen content. Therefore, the future development of antibacterial phototherapy MNMs should focus on these aspects.

Sonodynamic therapy (SDT) is a new technology that utilizes the interaction between ultrasound (US) and sonosensitizers to produce cytotoxic reactive oxygen species (ROS) and kill bacteria. Compared to light triggering, US has superior tissue penetrability, making it a promising approach for deep-sited infections. For instance, Wu et al. developed multifunctional US-responsive MNMs (RBC-HNTM-Pt@Au) for the treatment of MRSA-infected osteomyelitis [89]. RBC-HNTM-Pt@Au consists of a gold nanorod (AuNRs)-actuated single-atom-doped porphyrin metal–organic framework (HNTM-Pt@Au) and red cell membrane (RBC). Under US irradiation (1.5 W cm^−2^, continuous, 1 MHz), RBC-HNTM-Pt@Au can be directionally propelled at speeds of 0.77 mm/s, mainly attributable to the asymmetric structure and steady streaming stress generated by US. With its strong electron-trapping and oxygen adsorption capacity, RBC-HNTM-Pt@Au displayed excellent ultrasonic sensitization activity and antibacterial performance. It achieved an antibacterial efficiency of 99.9% against MRSA after just 15 min of US irradiation. In an MRSA-infected osteomyelitis model, the US + RBC-HNTM-Pt@Au group successfully eradicated the bacteria through 30 min of effective SDT after 4 weeks of treatment, proving the potential of US-driven MNMs for anti-infection therapy in deep tissue.

## Preclinical application

Superficial tissue infections and implant infections are the two major challenges in the field of antibacterial therapy [90]. The former involves topical application to increase drug concentration in the lesion, but it often results in the development of drug resistance [91]. Conversely, the latter necessitates prolonged antibacterial therapy duration and dose due to difficulties in removing bacteria colonizing the graft, resulting in increased side effects and recurrence [92]. However, local use of MNMs effectively eliminates colonizing bacteria without inducing drug-resistant strains, which addresses a crucial gap in antibacterial treatment. Fortunately, the topical application of nanomedicines may be approved for clinical use earlier than systemic administration (usually oral or intravenous), primarily due to concerns about potential systemic biological toxicity [93].

### Superficial tissue infections

Skin tissue is mammals’ first line of defense against bacterial invasion. When skin tissue is damaged, bacteria may attach and proliferate on its surface, leading to wound infections [94]. Bacteria create membranes at the site of infection and resist penetration by small molecules of antibiotics [95]. Therefore, breakthroughs in biological barriers are of great importance for anti-bacterial infection treatment [96, 97].

**Fig. 5:**
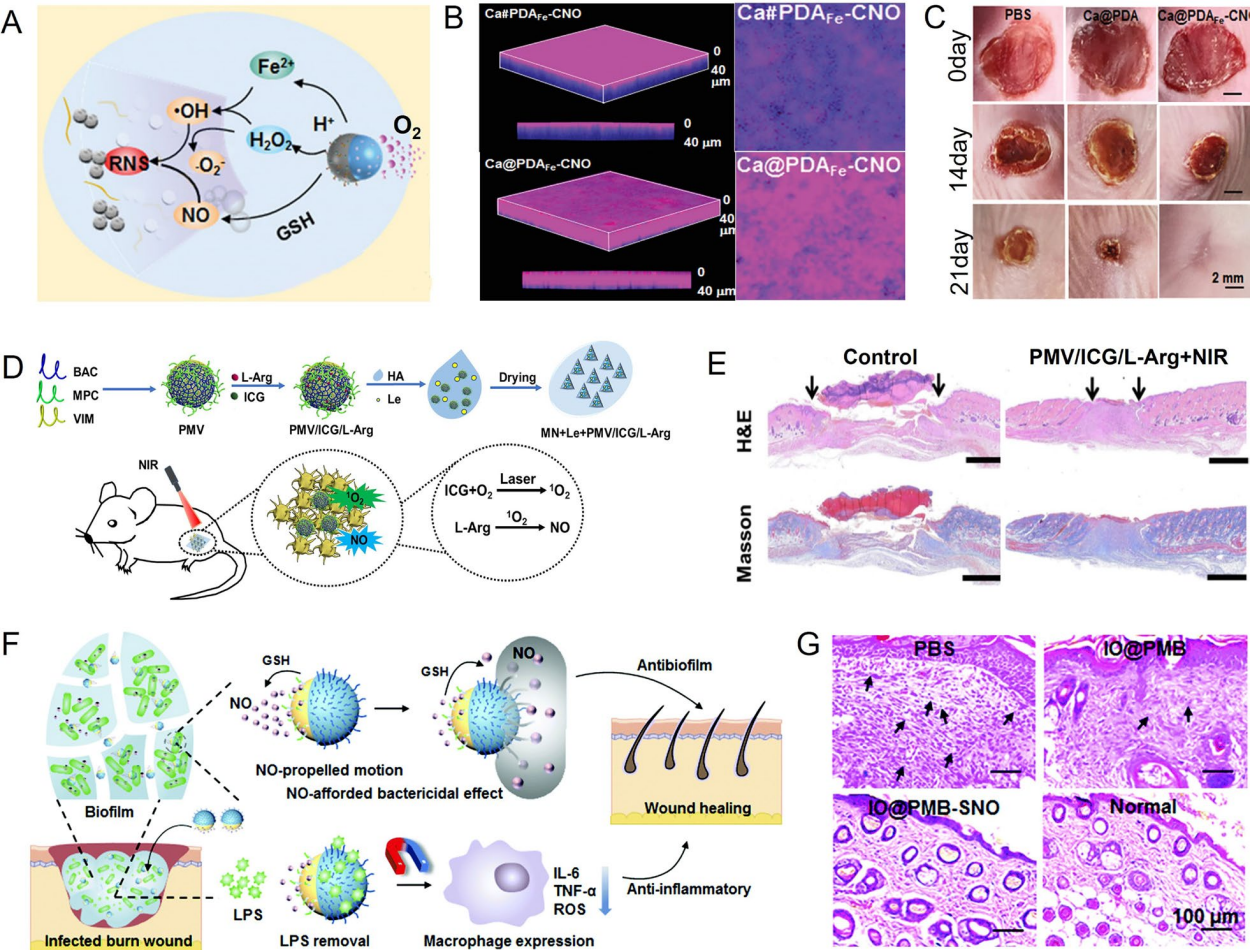
MNMs for the treatment of superficial tissue infections. **A** The driving and therapeutic mechanism of Ca@PDA_Fe_-CNO NPs. **B** Penetration biofilm performance of propelling and non-propelling nanomotors. **C** Visual images of *S. aureus*-infected wounds after treatment for 21 days. **D** The mechanism of microneedle patches used in wound antibiofilm therapy. **E** H&E staining and Masson staining of wound tissues in different treatment groups. **F** Schematic illustration of IO@PMB-SNO for antibacterial and anti-inflammatory therapy. **G** H&E staining images of wound tissues after different treatments for 12 days. **A–C** Reprinted with permission [98]. Copyright 2022, WILEY–VCH. **D, E** Reprinted with permission [100]. Copyright 2023, Elsevier. **F, G** Reprinted with permission [104]. Copyright 2022, The Royal Society of Chemistry

Recently, novel anti-bacterial strategies based on MNMs have achieved outstanding effectiveness in the treatment of superficial tissue infections. For example, Li et al. prepared a pH-responsive self-propelled nanomotor (Ca@PDA_Fe_-CNO) by grafting cysteine-NO (CNO) onto Janus CaO_2_ NPs partially coated with polydopamine (PDA) layers [98]. This was done in order to enhance biofilm infiltration and promote antibiofilm destruction. The nanomotors generated reactive nitrogen species (RNS) through a series of cascade reactions in the acidic biofilm microenvironment (BME). These RNS were able to destroy bacterial walls, bacterial membranes, and DNA. The acid-labile decomposition of CaO_2_ generated O_2_ from one side of the Janus NPs to propel the nanomotors diffusion in biofilms (Fig. 5A). In contrast, non-propelling nanoparticles (Ca#PDA_Fe_-CNO) showed significantly lower diffusion efficiency throughout the *S. aureus* biofilm matrix, as observed by confocal laser scanning microscope (CLSM) (Fig. 5B). Self-propelled Ca@PDA_Fe_-CNO diffused with a 12.1-fold increase in efficiency compared to non-propelling nanoparticles. Additionally, the efficiency of the antibacterial membrane was increased by 11.1 times, leading to the death of more than 99% of the bacteria. Interestingly, low levels of NO (intermediate products) released by nanomotors were found to enhance endothelial cell migration and collagen deposition. These effects accelerated wound healing and facilitated the repair of skin defects (Fig. 5C).

In addition, wound exposure can lead to persistent bacterial infestation, hindering wound recovery [99]. To address this issue, Mao et al. designed a microneedle patch loaded with nanomotor for the effective removal of biofilms and prevention of bacterial reinfections (Fig. 5D) [100]. The microneedle (MN) patches, referred to as MN + Le + PMV/ICG/L-Arg, consist of three components: sodium hyaluronate (HA) as a physical barrier, luteolin (Le) as a biofilms inhibitor and surface antibacterial agent, and nanomotors containing photosensitizer ICG and NO donor L-arginine (L-Arg) as deep antibacterial agents. When the microneedle patches are applied to an infected wound and irradiated with NIR light, the thermal gradient generated by ICG and the NO generated by L-Arg enable the nanomotors to form channels within the bacterial biofilms. As a result, the microneedle patches exhibited the triple effect (NO/PDT/PTT), which facilitates to achieve biofilm removal, antibacterial activity, and repair of infected wounds (Fig. 5E).

In addition to bacterial infections, persistent inflammation can also delay wound healing and lead to secondary infections [101, 102]. Lipopolysaccharide (LPS) endotoxins secreted by bacteria play a crucial role in chronic inflammation by interacting with toll-like receptors and activating an inflammatory response [103]. Thus, it is essential to consider effective endotoxin removal when attempting to kill bacteria. Li et al. developed IO@PMB-SNO, a GSH-responsive and magnetic recyclable nanomotor, as a solution to enhance biofilm infiltration, bacterial destruction, and endotoxin clearance, thereby accelerating wound healing (Fig. 5F) [104]. The nanomotors were created by grafting polymyxin B (PMB) and thiolated nitric oxide (SNO) donors onto partially coating Fe_3_O_4_ NPs with PDA layers. During the initial stages of treatment, the IO@PMB-SNO nanomotors respond to elevated GSH levels in the biofilms, releasing NO, resulting in self-propelled motion and non-antibiotic destruction of the biofilms and bacteria. The experimental results showed that the antibacterial rate of IO@PMB-SNO reached 93.6% and the biofilm dispersion efficacy was as high as 88.1%, respectively. Subsequently, PMB adsorbs LPS present on the surface of Gram-negative bacteria and released during bacterial division. Finally, the magnetic IO@PMB-SNO nanomotors, with adsorbed LPS, are removed from the infected wounds under a magnetic field. This resulted in an 89.5% reduction in endotoxins levels at the infected site. Thus, in burn wounds infected with *P. aeruginosa*, the IO@PMB-SNO treatment group displayed significant improvements in biofilm infiltration, bacterial killing, and skin tissue repair (Fig. 5G).

Antibacterial MNMs fill the gap in the treatment of superficial tissue infections, offering diversity and versatility in antibacterial treatment. This significantly enhances the efficiency of drug-resistant bacteria treatment. Clinical practice no longer recommends the local application of antibiotics due to the potential for inducing drug resistance. Therefore, antibacterial MNMs have emerged as a viable alternative for treating superficial tissue infections, exhibiting promising market prospects.

### Implants infections

**Fig. 6:**
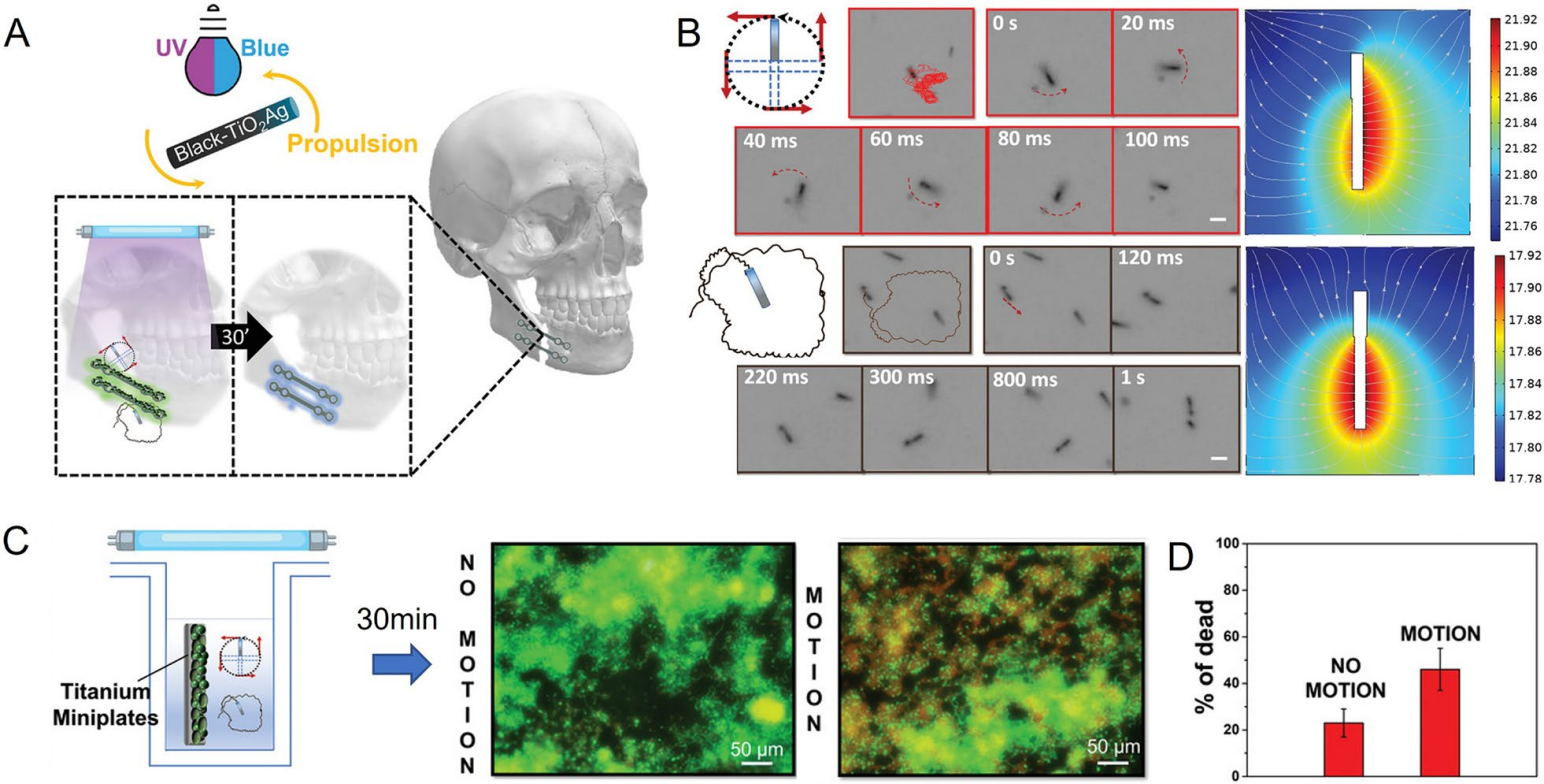
MNMs for the treatment of implants infections. **A** Schematic illustration of B-TiO_2_/Ag nanomotors for removing biofilm from facial titanium miniplates. **B** Trajectories images, time-frame images and H^+^ gradient spatial distribution images of B-TiO_2_/Ag nanomotors after exposure to UV-light irradiation in 0.1% of H_2_O_2_. **C** Live/dead cell fluorescent images of biofilm onto titanium facial implants after treatment with static and moving B-TiO_2_/Ag nanomotors. **D** Percentage of dead bacteria cells after different treatment. **A–D** Reprinted with permission [111]. Copyright 2022, WILEY–VCH

Implantable devices, such as stainless-steel/titanium nail, heart valves, cardiac pacemaker, and artificial lenses, have been used for the past half-century to treat various illnesses and improve the quality of life for many patients [105–107]. However, their introduction into the body creates a potential risk of microbial colonization and infection [108, 109]. In fact, the mortality rate of implant infections is significantly higher than that of organ infections due to the ease of bacterial biofilm colonization and the challenges in clearing them with antibiotics [110]. Fortunately, antibacterial MNMs provide a promising solution to implant infections. Pumera et al. have proposed a novel approach using light-driven self-propelled tubular nanomotors (Ag/B-TiO_2_), which are based on Black-TiO_2_ decorated with Ag NPs by physical deposition, to degrade bacterial biofilm growth on commercial facial titanium miniplate implants (Fig. 6A) [111]. When exposed to visible lights/UV, the B-TiO_2_ side catalyzes H_2_O and H_2_O_2_ to produce ROS (OH· and O_2_^·−^), while the Ag side reduces H^+^ and H_2_O_2_ to H_2_O. This process creates a gradient and a local electric field, which allows the nanomotors to propel themselves via the self-electrophoretic mechanism (Fig. 6B). The ROS generated by photocatalysis and Ag^+^ produced by oxidation then act to kill bacteria and remove biofilms. In experiments conducted on titanium fixation plates colonized by MRSA, Ag/B-TiO_2_ was able to remove approximately 40% of the biofilm and eradicate 36% of the bacteria after being exposed to UV light for 30 min (Fig. 6C, D). This breakthrough offers a promising new avenue for implant bacteria colonization therapy.

In conclusion, there is still a long way to go before antibacterial MNMs can be successfully translated into in vivo application, despite their promising results in preclinical studies. Researchers have acknowledged the importance of safety design in the movement of MNMs within the human body. When MNMs are introduced into the human environment, several factors need to be taken into consideration, such as the biosafety of their components (including biocompatibility, potential immune response, and their ability to be eliminated from the body), as well as the biosafety of the driving system. Therefore, significant efforts have been made in four main directions. Firstly, researchers have developed biomimetic MNMs by combining natural biological materials, like red blood cell membranes or platelet membranes, with synthetic materials, resulting in good biocompatibility and no immune response [112, 113]. Secondly, MNMs have been designed to be biodegradable or self-destructive, such as biodegradable polymer nanomotors or acid-powered Mg-based micromotors, thus avoiding potential biological toxicity caused by their accumulation [114, 115]. Thirdly, ideal MNMs for in vivo applications should utilize harmful components present in the infected microenvironment as a driving substrate, with the reaction products being beneficial or harmless to the human body. For instance, a bifunctional nanozyme has been developed with peroxidase-like and catalase-like activity, which can decompose toxic H_2_O_2_ into strongly oxidizing hydroxyl radicals (·OH) to prevent bacterial infection and generate abundant O_2_ as potential driving power [116]. It is our opinion that the most suitable initial application of MNMs in human clinical settings would be in superficial tissue infections, as this could minimize the potential risks associated with systemic applications.

## Summary and outlook

In this review, we introduce and summarize the driving mechanism and antibacterial principle of MNMs in detail (Table 1). Antibacterial MNMs effectively break through bacterial biofilms and can be combined with new antibacterial strategies to treat multi-drug resistant bacteria. Two difficult problems in the field of anti-infection, superficial tissue infections and implant infections, are then discussed in the application of MNMs.

**Table 1:** Summary of advantages and disadvantages of MNMs based on different propulsion mechanisms

Propulsion type	Substrates or energy	Propulsion mechanisms	Advantages	Disadvantages	References
Chemical propulsion	Gastric acid, l-arginine, H_2_O_2_, glucose, urea, etc	Bubble propulsion, self-diffusiophoresis and self-electrophoresis	Chemical-propelled MNMs are simple to operate, do not require external actuation systems and can produce high movement speeds. Adapted for specific diseases such as gastrointestinal and urinary system diseases, because they can respond to special ingredients in the disease environment (such as gastric acid, urea, etc.)	Chemical-propelled MNMs usually exhibit random locomotion and a lack of directionality. In addition, continuous fuel requirements and potential risks of gas generation limit in vivo applications	[48, 49, 57, 58, 68, 98, 100, 104]
External physical fields propulsion	Light energy	Local thermophoresis	By changing the intensity or lighting direction, both velocity and direction of MNMs can be manipulated. Moreover, Photoactive MNMs can absorb light energy and trigger PDT, PTT and PCT	The tissue penetration depth of NIR is low, only 1–2 cm, which is not suitable for deep tissue application. Prolonged exposure may result in potential skin tissue damage	[55, 62, 81, 88, 110]
	Ultrasound waves	Ultrasonic forces	Ultrasonic driven MNMs exhibits good directionality, strong penetration ability and outstanding biocompatibility. Furthermore, with tunable acoustic parameters (e.g., frequency, voltage), these MNMs usually demonstrate powerful propulsion	Compared with photoactivated nanomaterials, the types of acoustic activated nanomaterials are relatively few and need to be further developed	[89]
	Magnetic fields	Magnetic force	Magnetically driven MNMs has good controllability and navigation, which can achieve remote, precise, and multi-degree of freedom motion control. Topical application can be recycled to avoid internal accumulation	Driving magnetic MNMs at scales of several microns is difficult owing to scaling laws. In addition, the use of magnetic MNMs will limit some clinical examinations, such as MRI	[80]

Despite the excellent therapeutic effects of antibacterial MNMs, there are several areas of concern. Firstly, the direction of motion of MNMs is difficult to control, resulting in low utilization efficiency. Secondly, the driving force of the MNMs is not long-lasting due to the biological substrate content in vivo and the attenuation of light in the tissue. Thirdly, while antibacterial strategies based on MNMs do not induce antibiotic resistance, it is worth noting that smart and formidable bacteria may develop adaptive mechanisms over time. For example, bacterial resistance to silver nanoparticles has been reported [117]. Fourthly, while most materials are considered biocompatible, they may still be severely immunogenic and have maximum tolerance. Finally, MNMs will still face the challenge of medical ethics in the process of clinical transformation.

Future efforts should focus on several aspects. Firstly, directional control of MNMs is a primary focus of research, and there are various methods to achieve this. Magnetic MNMs have the advantage of being able to guide motion through an external magnetic field. On the other hand, glucose oxidase-based MNMs use the concentration difference between the two sides of the biological barrier, such as the blood–brain barrier, to achieve directional driving [118]. Secondly, synthetic MNMs could use adenosine triphosphate (ATP) as a power source, inspired by intracellular kinesin, to reduce the production of harmful gases and maintain a stable internal environment. Thirdly, it is crucial to study the resistance mechanisms of bacteria to nanomaterials, particularly in efflux pump, redox, and heat resistance [119]. Fourthly, the selection of nanomaterials may be particularly important for future applications in vivo. Notably, the application of biodegradable materials will be a trend in the future development of medical MNMs to avoid biotoxicity caused by the accumulation of metals in the body. Fifthly, designing MNMs with imaging function has significant advantages in guiding treatment, detecting treatment effects, and tracking biological metabolism, which will promote the visualization of anti-infection therapy. MNMs-based optical microscopy imaging, fluorescence imaging (FI), magnetic resonance imaging (MRI), radionuclide imaging (RI) and photoacoustic computed tomography (PACT) will provide more information from different perspectives to guide antibacterial therapy [56, 120].

Finally, research on the biosafety of MNMs will be the collaborative research challenge for experts in micro/nanoscience, materials science, physics, chemistry, engineering science, life science, and medical fields. Biosafety is considered one of the most crucial factors in transformative medicine [121]. In this regard, artificial intelligence (AL) and machine learning (ML) have made significant breakthroughs in toxicology studies of nanomaterials [122, 123]. In the process of clinical transformation, MNMs will face the challenge of medical ethics. This is a main concern addressed by “nanoethics”, which primarily focuses on ethical issues related to nanoscience and technology. Specifically, it deals with biohybrids and medical applications of advanced nanomaterials [124–126]. To effectively manage the potential health and environmental risks associated with nanomaterials, early development of policies and regulations is necessary. This proactive approach will also contribute to the achievement of clinical conversion [127, 128].

In conclusion, while progress has been made, there is still a long way to go from in vitro research to in vivo application. This review aims to stimulate further development of MNMs in the field of antibiotic therapy for the benefit of patients.

## Data Availability

We have included 5 figures (Figs. 2, 3, 4, 5 and 6) from previously published literature with required copyright permission from the copyright owners. We have mentioned this in the manuscript with appropriate citations.

## Abbreviations

AL: Artificial intelligence
AMPs: Antimicrobial peptides
ATP: Adenosine triphosphate
BME: Biofilm microenvironment
CLA/CLR: Clarithromycin
CLSM: Confocal laser scanning microscope
CNO: Cysteine nitric oxide
DMSNs: Dendritic mesoporous silica nanoparticles
EPS: Extracellular polymeric substances
FI: Fluorescence imaging
GSH: Glutathione
HA: Hyaluronate
ICG: Indo cyanine green
L-Arg: L-arginine
LPS: Lipopolysaccharide
MDR: Multi-drug resistant
ML: Machine learning
MN: Microneedle
MNMs: Nano/micromotors
MRI: Magnetic resonance imaging
MRSA: Methicillin-resistant *S. aureus*
NBs: Nanobottles
NDs: Nanodendrites
NO: Nitric oxide
NPs: Nanoparticles
NIR: Near-infrared
PA: Photoacoustic
PACT: Photoacoustic computed tomography
PCT: Photocatalysis therapy
PDA: Polydopamine
PDT: Photodynamic therapy
PEDOT: Poly(3,4-ethylenedioxythiophene)
PLGA: Poly(lactic-co-glycolic acid
PMB: Polymyxin B
PTT: Photothermal therapy
RI: Radionuclide imaging
RNS: Reactive nitrogen species
ROS: Reactive oxygen species
SNO: Thiolated nitric oxide
TAPP: 5,10,15,20-Tetrakis(4-aminophenyl)porphyrin
UV: Ultraviolet
